# Is All Dating Violence Equal? Gender and Severity Differences in Predictors of Perpetration

**DOI:** 10.3390/bs10070118

**Published:** 2020-07-20

**Authors:** Gabriela Ontiveros, Arthur Cantos, Po-Yi Chen, Ruby Charak, K. Daniel O’Leary

**Affiliations:** 1 Department of Clinical Psychology, The University of Texas Rio Grande Valley, 1201 W University Dr, Edinburg, TX 78539, USA; arthur.cantos@utrgv.edu (A.C.); poyi.chen@utrgv.edu (P.-Y.C.); ruby.charak@utrgv.edu (R.C.); 2 Department of Psychology, Stony Brook University, 100 Nicolls Road, Stony Brook, NY 11794, USA; daniel.oleary@stonybrook.edu

**Keywords:** teen dating violence, TDV, physical perpetration, severity, emotion regulation, attachment, impulsivity, interparental violence, ACEs

## Abstract

The present study assesses the extent of perpetration of physical violence in predominately Hispanic high school students in the Rio Grande Valley, Texas. The relationship between adverse childhood experiences, exposure to interparental violence, attachment, emotion regulation, and impulsivity on two distinct, mutually exclusive, categories of severity of physical teen dating violence (TDV) perpetration is further explored. Participants completed self-report measures as part of a larger, anonymous web-based questionnaire. Two categories (i.e., minor/moderate and severe) were created to discern the contextual variables associated with different levels of severity of physical violence perpetration by males and females. Eight-hundred and twenty-nine 14- to 18-year-old adolescents from four different high schools participated in the study, of whom 407 reported having been in a dating relationship in the last 12 months. The results demonstrate that when only the most severe item of TDV is taken into consideration, the rates of violence perpetration by males and females are almost equal and remarkably lower than those reported in the literature. However, when the assessment includes minor/moderate levels of violence, such as pushing, the rates of violence perpetration by females are twice those of males and are consistent with those reported in the literature. Furthermore, different variables are associated with different levels of severity of violence perpetration. The results support approaches that emphasize the need to take the context of the violence into consideration, since all levels are not equal. The need to take the severity of violence into account in studies assessing dating violence is highlighted.

## 1. Introduction

Adolescence, the transitional stage between childhood and adulthood, is typically marked with elements of insecurity, immaturity, and faltering emotion regulation [1]. In this developmental period, courtship behavior develops, and the risk of teen dating violence (TDV) perpetration and/or victimization emerges [2]. Various studies have indicated that violence towards a partner peaks in adolescence [3,4]. A 10-year longitudinal study [5] on men’s aggression over time found a downward linear relationship between age and physical violence perpetration, regardless of changes in relationships. Using data from various cross-sectional studies, O’Leary [6] estimated that physical aggression towards an intimate partner increases considerably between the ages of 15 and 25 years and declines thereafter. Taken together, data from longitudinal and cross-sectional designs emphasizes the importance of studying violence at this stage of development. Consequently, the study of dating violence in adolescence has the potential to facilitate an understanding of risk factors for intimate partner violence (IPV) in adulthood [7,8].

Violence in dating relationships encompasses many forms of abuse, including threatening behavior, physical aggression, sexual abuse, and verbal or emotional abuse. Acts of abuse range from spreading rumors to physically harming or coercing in an attempt to have sex [7,8,9]. Operationalization of the different types of TDV is seen in commonly used scales, such as the Revised Conflict Tactics Scales-2 (CTS2) [10] and the Conflict in Adolescent Dating Relationships Inventory (CADRI) [9]. Each scale uses multiple items to generate different subscales for the distinct forms of abuse, recognizing their equal contributions to TDV as a whole [3,11]. In contrast, some studies use single items to assess for abuse (e.g., physical abuse) embedded within a questionnaire [12]. Nevertheless, the prevalence rates of physical teen dating violence are disparate. In a U.S. national sample of 3614 adolescents that only considered serious dating violence, 0.8% reported having experienced physical assault [13]. Another study using the CADRI to measure TDV found that among 1653 middle school students in a dating relationship, 32% reported perpetrating physical TDV [14]. More recently, a meta-analysis on the review of prevalence rates of physical violence from 96 studies reported rates ranging from 1% to 61% [15]. In addition, this same review described gender differences in physical violence perpetration. Specifically, girls reported perpetrating more physical violence than boys (25% vs. 13%) [15]. When analyzing youth who identified with an ethnic minority, girls who differed in culture from the majority cultural group in the studies were more likely to perpetrate physical TDV [15]. Prevalence rates of physical TDV specifically for Latino/a adolescents are dissimilar as well. Due to differences in sampling methods, rates for Latino/a youth range between 5% and 44% [16,17,18,19,20]. A national study on Latino youth conducted by Cuevas, Sabina, and Bell (2014) found that TDV increases with age (at around 14 years), with boys more likely to experience violence (18.35%) [16]. A second study assessing the patterns of violence among Latino youth found that TDV prevalence rates for girls were higher than for their counterparts; girls’ prevalence rates ranged from 11.2% to 19.8%, while boys’ rates ranged from 6.5% to 10.6% [19]. Regarding physical dating violence, in the TDV literature, youth who identify as Latino/a have been found to report higher rates than those who identify as White non-Hispanic [21,22].

Overall, the literature on TDV supports the idea that violence among youths has become a pervasive health problem [13,14]. Nevertheless, the high variability in prevalence rates make it difficult to assess contextual variables that can be associated with varying degrees of physical perpetration. Different types of assessments result in different rates of perpetration and victimization. This study sought to combine research designs that include either multi-item subscales assessing for varying severities of physical TDV perpetration or single-item assessments of the most severe type of physical TDV to provide prevalence rates at differing levels of severity. In addition, it attempted to identify contextual variables associated with perpetration of physical TDV by male and female adolescents, at different severity levels for high school students identifying as Hispanic or Latino/a.

### 1.1. Adverse Childhood Experiences and Exposure to Interparental Violence

In the dating violence literature, one of the most common studied risk factors for perpetration and victimization is exposure to traumatic experiences during childhood [23,24]. First described by Felitti and colleagues in their seminal ACE study of 1998, adverse childhood experiences, or ACEs, encompass three forms of traumatic exposure: abuse, neglect, and household challenges [25]. Abuse variables include emotional, physical, and sexual abuse; neglect encompass emotional and physical neglect; and household dysfunction variables comprise interparental violence, parental separation or divorce, and household substance use [25]. Together, these three categories of ACEs have been shown to have deleterious effects on youth. A 2009 national survey by the Office of Juvenile Justice and Delinquency Prevention found that more than 60% of children younger than 17 years had been exposed to violence within that year, whether indirectly by being a witness or directly by being a victim [26]. Approximately 10% of children endured either physical abuse, emotional abuse or neglect, and 9.8% witnessed one family member assaulting another [26]. The 2015 National Survey of Children’s Exposure to Violence (NatSCEV) found that within the past year 57.3% of children younger than 17 years had been victims of any kind of assault, 15.2% had been victims of any maltreatment, 24.5% had witnessed any type of violence, and 8.4% had witnessed a family member assaulting another [27].

A growing body of literature has explored the health outcomes that result when a higher number of ACEs are reported and their subsequent negative compounding effect across the lifespan [25]. In addition, prior studies have assessed the impact of ACEs on adolescent behavior, specifically physical TDV perpetration as a consequence of ACEs. A 2019 study measuring the effects of ACEs on violence perpetration found a positive association with perpetration of TDV and exposure to ACEs [28]. In this study, boys reported being exposed to a greater number of ACEs than girls; however, both were at increased odds for perpetration of TDV with more exposure to various ACEs [28]. Longitudinal designs, and limited research, demonstrate a more consistent link between ACEs and future perpetration of TDV, including exposure to ACEs and violence within adolescent dating relationships [29] and future risk of intimate partner violence [30]. A study on approximately 37,000 ninth grade students found that the most common reported ACEs were verbal abuse, physical abuse, parental substance abuse, witnessing interparental violence, having a parent incarcerated, and sexual abuse [31]. When students were divided into subgroups based on bullying victimization, perpetration, or both, 40% of students comprising the perpetrator only group reported ACEs in the family [31]. Regarding gender, an increase of 0 to 6 ACEs was associated with a 34% increase in probability of physical bullying perpetration in girls and a 64% increase of bullying perpetration in boys [31]. Compared with youth who reported no instances of ACEs, youth with ACEs were more at risk of being victims of school-based abuse, perpetrators of school-based abuse, or both [31]. However, it is not very well understood how adverse experiences in childhood and maltreatment lead to TDV. The household dysfunction category of ACEs includes exposure to interparental violence. Various theories have attempted to explain the relationship between witnessing violence in the family during childhood and later perpetration and victimization of violence. 

A popular theoretical framework used to explain “the cycle of violence” is Bandura’s social learning theory [32]. It posits that children model behavior they observe from their family (i.e., their parents) and this behavior is reinforced throughout adolescence and continues in the latter stages of life as a method of conflict resolution [33]. Moreover, Riggs and O’Leary (1989) relied on social learning theory to formulate a background-situational model for dating violence [34]. In an attempt to understand and explain perpetration and victimization of such violence, this model sought to describe the associations among background variables (i.e., exposure to interparental violence), situational variables (i.e., alcohol or drug use, problem-solving skills, relationship length, etc.), and dating violence [34}. A notable longitudinal study that followed 543 children for 20 years found the social learning model of partner violence to hold true [35]. Exposure to interparental violence remained a potent predictor for later partner abuse perpetration and posed the greatest independent risk for partner violence victimization compared to physical abuse and adolescent conduct disorder [35]. The significant relationship between exposure to interparental violence and later physical TDV perpetration and victimization has been explored and supported in various studies [36,37].

### 1.2. Attachment

Further research on mediators/moderators of dating violence has shown that adverse childhood experiences and exposure to interparental violence can lead to the development of maladaptive attachment styles [38]. Attachment theory, first introduced by Bowlby in 1982, posits that infants are born with an intrinsic drive to seek proximity to caretakers in times of danger or threat [39]. Experiences with caregivers thus lead to expectations about future relationships and guide the behaviors in those [38,39]. Individuals with a secure attachment style display independence and are comfortable with emotional proximity and intimacy; in contrast, insecurely attached or anxious individuals yearn for intimacy, display a fear of rejection, and oftentimes are characterized by feelings of jealousy. Moreover, individuals with avoidant or dismissing attachment styles circumvent intimate relationships and find discomfort in closeness [39]. Although research on attachment and its association with violence perpetration and victimization has been studied in the context of adult relationships, there has been limited research on its association with adolescent relationships.

A study using a 10-month longitudinal design found a positive relationship between TDV perpetration in middle- and high-school-age youth and attachment anxiety. Adolescents who reported more attachment anxiety were more likely to report more experiences of TDV perpetration 10 months later [40]. In a study of undergraduate students between the ages of 18 and 25, attachment anxiety and attachment avoidance were not significant predictors of dating violence perpetration in men; however, in women, attachment anxiety, and not attachment avoidance, was predictive of dating violence perpetration [41]. In a third study in a predominately African American sample it was found that for those with a secure attachment pattern, their history of childhood maltreatment was not a significant risk factor of dating violence [42]. In addition, the same study found a moderating role of avoidant/dismissing attachment in the pathway from exposure to maltreatment in childhood to dating violence victimization [42]. Although not using an adolescent sample, a study by Babcock, Jacobson, Gottman, and Yerington (2000) found that dismissing husbands were more likely to perpetrate violence if the wife was defensive and preoccupied men were more likely to perpetrate violence if they noticed their wife’s withdrawal [43]. Based on the findings, they posited that men who were categorized as preoccupied were more likely to perpetrate violence as a result of fear of abandonment and men who were categorized as dismissing were more likely to use instrumental violence to assert control [43]. Collectively, the few studies examining the role of attachment as a predictor of TDV perpetration and victimization and the literature on adult relationships and attachment are suggestive of an association between maladaptive attachment styles and TDV perpetration and victimization.

### 1.3. Emotion Dysregulation and Impulsivity

Attachment styles have also been shown to be related to emotion regulation and various research studies in adults have linked the two [44]. Drawing from contemporary views on attachment, individuals with secure attachment styles tend to be more self-aware regarding their emotions and better able to regulate both positive and negative affect [44,45]. Conversely, individuals high in insecure-anxious attachment are characterized by deep worry and fear, especially with regard to imagined abandonment [44]. Last, individuals with dismissing attachment have been found to reduce their distress so as to not trigger their attachment systems [44]. Early research on the emotion of children and adolescents indicated that in the adolescent stage, more intense emotions are experienced [46]. Various theories for this phenomenon have been posited, such as hormonal and neurological changes that are characteristic of this developmental stage [46].

While emotion dysregulation has been investigated as a potential risk factor for dating violence perpetration in adults [26,27], little is known about the relationship between emotion dysregulation and violence perpetration in teen dating relationships. A study conducted on a community sample of youths sought to analyze borderline personality pathology features (including emotion dysregulation and impulsive behavior) in adolescents as it related to TDV perpetration and victimization [47]. The findings showed that borderline features displayed a strong association with victimization and perpetration of TDV, and this association was further affected by gender [47]. Girls were more likely to be victims of TDV, but when violence was more severe (i.e., only physical and sexual abuse), they were more likely to be both perpetrators and victims [47]. This was the first study to provide preliminary evidence that the role of borderline personality pathology features in intimate partner violence perpetration and victimization can in fact be expanded to adolescents.

Drawing on findings from literature on adult intimate partner violence, it is possible that the same mechanism of poor emotion regulation and impulsivity can be associated with perpetration and victimization of violence in adolescent dating relationships as well. Hence, the present study sought to analyze the associations among adverse childhood experiences, exposure to interparental violence, attachment styles, emotion dysregulation, impulsivity, and physical TDV perpetration in a community sample of predominately Hispanic/Latino/a High School adolescents from the Rio Grande Valley, Texas.

### 1.4. The Present Study

Much of the literature on dating violence has relied on the background-situational model posited by Riggs and O’Leary (1989) which proposes that factors such as exposure to violence in the family and prior aggression will increase the likelihood that an individual will perpetrate violence in a dating relationship [34]. Moreover, much of what we know about possible predictors of TDV stems from studies consisting of White non-Hispanic youth [17]. To date, there is sparse literature on the experiences of Latino/as and TDV, and no current study has analyzed the experiences of youth from the Rio Grande Valley, Texas, a predominantly Hispanic population (92%) [48]. Furthermore, an important gap in the literature of adolescent relationship violence is the failure to discriminate between more and less severe items in scales assessing prevalence rates of physical TDV perpetration, and the factors predicting such violence. Thus, contextual variables highlighted as being associated with physical TDV perpetration cannot be discerned from context (i.e., severity of physical TDV perpetration). This study aimed to identify predictors of physical TDV perpetration when all items of a physical TDV subscale were used, then further by creating two mutually exclusive categories for items gauging minor/moderate levels of severity of physical TDV perpetration and those that measure the most severe levels.

Accordingly, the first aim of the present study was to examine the differential prevalence rates of physical TDV in a high school sample of males and females in the Rio Grande Valley, Texas, based on all four items of the CADRI, and two categories (i.e., 1: only the minor and moderate severity; 2: only the most severe). We focus on physical TDV perpetration because it has received the most attention in the literature, and oftentimes leads to the most severe consequences that can likely result in serious injury and emotional outcomes [49].

The second aim of the present study was to investigate the following contextual variables: adverse childhood experiences, exposure to interparental violence, attachment, emotion dysregulation, and impulsivity, in an attempt to examine what correlates would be associated with physical TDV at each category of severity. In line with Bandura’s social learning theory [32] and Rigg and O’Leary’s background-situational model [34], we expected that higher instances of ACEs and exposure to interparental violence would be positively related to physical TDV perpetration across levels of severity (hypothesis 1). In addition, we hypothesized that attachment would be associated with physical TDV perpetration. Specifically, a dismissing attachment style (hypothesis 2) would be associated with perpetration in male adolescents, and a fearful attachment style in female adolescents (hypothesis 3). Further, various studies have noted that childhood adversities affect developmental and formative years, and in the absence of care or appropriate parenting skills, the developing child fails to learn to regulate their emotions in the face of distress [50]. Thus, we hypothesized that impulsivity and emotion dysregulation would moderate the association between ACEs, interparental violence, and physical TDV perpetration, and these relationships would be dependent on gender and levels (hypothesis 4).

## 2. Materials and Methods

### 2.1. Participants

Study participants were selected from four public high schools in Hidalgo County in the Rio Grande Valley, Texas which is positioned close to the United States–Mexico border and has a high concentration of local residents of Hispanic cultural backgrounds (91.2% Hispanics in Hidalgo County) [48]. The median income in Hidalgo County is $38,195, substantially below the average for the USA, and a 31% poverty rate [48]. The total sample comprised 829 14- to 18-year-old adolescents enrolled in grades 9–12. Recruitment was conducted at each of the respective high schools. A total of 407 adolescents (49.10%) reported being in a heterosexual dating relationship, regardless of duration, in the last 12 months and thus were eligible for analysis. The mean age of the sample was 16 (SD = 1.36). Thirty adolescents (4.0%) reported having had a same-sex relationship in the last 12 months. Although this number was too small for comparisons and consequently these adolescents were excluded from analysis, prevalence of physical TDV perpetration was reported.

### 2.2. Measures

#### 2.2.1. Demographic Information

Demographic characteristics were assessed using single items, and they included gender, age, grade, race/ethnicity, household size, primary language spoken at home, household income, and father and mother’s highest level of education. Gender was dichotomous: (1) for males and (2) for females. Due to the small proportion of ethnicities other than Hispanic/Latino, ethnicity was recoded into two categories: Hispanic/Latino (1) or Other (0). Estimated parents’ household income was assessed using twelve categories in $10,000 increments. Lastly, father and mother’s highest level of education was assessed using six categories: (1) Did not attend school; (2) Some high school; (3) High school graduate; (4) Some college; (5) College graduate; (6) Graduate school or Professional school.

#### 2.2.2. Physical Teenage Dating Violence Perpetration

Estimates of physical violence perpetration in dating relationships were measured using the Conflict in Adolescent Dating Relationships Inventory (CADRI) [9]. The CADRI, a 35-item instrument, assesses instances of five types of abuse within the last twelve months: threatening behavior, relational abuse, physical abuse, sexual abuse, and verbal/emotional abuse. The questions are paired; respondents first answer regarding their behavior towards a dating partner and then their dating partner’s behavior towards them. Items are rated on a four-point Likert scale system with the following distinctions: 0 = Never (0 times); 1 = Seldom (1–2 times); 2 = Sometimes (3–5 times); and 3 = Often (6 or more times). Regarding the study of dating violence, the CADRI and the Conflict Tactics Scale (CTS) are the most relevant; however, the CADRI has greater reliability and validity [9]. It has been shown to have strong internal consistency and adequate 2-week test-retest reliability for subscales (*r* = 0.68) [9]. Cronbach’s alpha coefficients were acceptable for verbal or emotional abuse (*α* = 0.82) and for physical abuse subscales (*α* = 0.83) [9].

**Figure 1:**
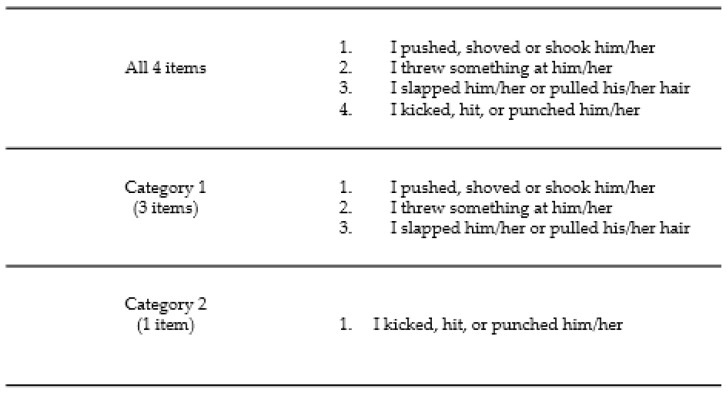
Categories derived from the CADRI physical abuse subscale.

For the present study, only the four items in the physical abuse subscale were used for analyses: “I pushed, shoved or shook her”, “I threw something at her”, “I slapped her or pulled her hair”, and “I kicked, hit, or punched her”. Dichotomizing the items allowed to assess for presence or absence of the abuse. Perpetrator status was based on endorsement of at least one occurrence of physical abuse (i.e., endorsing at least one of the four items). To assess for severity of the physical abuse, two mutually exclusive categories were created (Figure 1). Category 1 was classified as minor/moderate violence and included the first three items, excluding kicking, hitting, or punching (*n* = 46; female 37, male 9). If a participant endorsed one or more of the three items, they were assigned to category 1. Category 2 represented severe violence and included only kicking, hitting, or punching (*n* = 27; female 14, male 13). If a participant reported kicking, hitting, or punching, they were assigned to this category. Further, if a participant endorsed the most severe item (i.e., category 2) they were not counted at category 1 even if they had also perpetrated minor/moderate violence. Internal consistency for the current study was α = 0.83 for physical TDV.

#### 2.2.3. Adverse Childhood Experiences

In order to identify childhood experiences of abuse and neglect, the Adverse Childhood Experiences (ACEs) questionnaire was used [25]. The ACE scale consists of 17 items that assess the occurrence of an adverse event during the participants’ first 18 years of life. It contains two subscales: The Childhood Abuse subscale that contains eight questions about exposure to psychological, physical, and sexual abuse, and the Exposure to Household Dysfunction subscale that includes nine items about exposure to domestic abuse, substance abuse, criminal behavior in the household, and mental illness. Responses are dichotomous (0 = No, 1 = Yes), with higher scores indicating a greater number of adverse experiences in childhood. The ACEs questionnaire is a valid scale used to assess challenges in early years and previous studies have found that it has good to excellent test-retest reliability [25]. Cronbach’s alpha for the ACEs questionnaire was 0.80 in the present study.

#### 2.2.4. Exposure to Interparental Violence

Three dichotomous (0 = No, 1 = Yes) items were used to assess participants’ exposure to interparental violence. The following single items were asked: (1) Did you ever witness your father hit your mother?; (2) Did you ever witness your mother hit your father?; and (3) Did you ever witness both parents hit each other? The binary nature of the responses allowed to assess for presence or absence of exposure to interparental violence. Internal consistency for the present study was α = 0.83.

#### 2.2.5. Attachment

The Adolescent Relationship Scales Questionnaire (RSQ), a measure of attachment behavior, was used to describe aspects of the participants’ relationships and their patterns of attachment [51,52]. It consists of 17 items scored on a nine-point Likert scale system ranging from “not at all like me” to “very much like me”, and is used to indicate the degree to which a statement best describes the participant’s feelings. The instrument yields scores on four subscales: Secure, Fearful, Preoccupied, and Dismissing. The Secure subscale measures comfort with depending on others and intimacy. The Fearful subscale assesses anxiety over being hurt in the relationship and feeling insecure in the relationship. The Preoccupied subscale measures the extent to which the participant worries that people will not really care about them. The Dismissing subscale assesses lack of desire to be close to others, and emotionally distant and rejecting nature in intimate relationships. Cronbach’s alpha reported for the instrument is 0.60 and modest reliability was reported for the four subscales [51,52]. For the present study, Cronbach’s alpha ranged from α = 0.68 to α = 0.79.

#### 2.2.6. Emotion Dysregulation

The Difficulties in Emotion Regulation Scale (DERS) was used to provide a comprehensive measure of emotion dysregulation [53]. This 36-item self-report measure examines six different aspects of emotion dysregulation: non-acceptance of emotional responses (nonacceptance), difficulties engaging in goal-directed behavior (goals), impulse control difficulties (impulse), lack of emotional awareness (awareness), limited access to emotion regulation strategies (strategies), and lack of emotional clarity (clarity). Items are rated on a scale of 1 (“almost never”) to 5 (“almost always”), and higher scores indicate more difficulty in regulating emotion. All six subscales are moderately to strongly correlated and their internal consistency (Cronbach’s α) ranges from 0.80 to 0.89, with a total scale-internal consistency of 0.93. In addition, the DERS shows adequate test-retest reliability for a period of 4–8 weeks [54]. With its ability to assess multiple aspects of emotion regulation at one time, the DERS represents one of the most comprehensive available measures of emotion regulation [54]. In the present study, the Cronbach’s alpha for each of the five subscales in the DERS ranged from α = 0.75 to α = 0.88.

#### 2.2.7. Impulsivity

The Urgency, Premeditation (lack of), Perseverance (lack of), Sensation Seeking, Positive Urgency (UPPS-P) impulsive behavior scale was used to measure impulsive behavior [55]. The UPPS-P scale assesses five distinct dimensions of impulsive behavior in adolescents and adults. Consisting of 59 items, this self-report scale is not a measure of trait impulsivity, but rather reflects distinct personality traits that lead to impulsive-type behavior. Participants are asked to rate how much they agree or disagree with each statement using a 4-point Likert scale system of 1 (“agree strongly”) to 4 (“disagree strongly”). Higher scores on each of the five subscales indicate more impulsive behavior. The Negative Urgency subscale describes the tendency to act rashly under extreme negative emotions. The Lack of Premeditation subscale describes the tendency to act without thinking. The Lack of Perseverance subscale describes the inability to remain focused on a task. The Sensation Seeking subscale describes the tendency to seek out novel and thrilling experiences. The Positive Urgency subscale describes the tendency to act rashly under extreme positive emotions. Internal consistencies (Cronbach’s α) range from 0.82 to 0.92 [55]. For the present study, Cronbach’s α ranged from 0.72 to 0.93.

### 2.3. Procedure

#### 2.3.1. Recruitment Procedure

Recruitment of participants was conducted in classes with required attendance. At each of the four high schools, an administrative officer randomly selected the first classes available in a non-core subject area (i.e., elective classes) by choosing from a list. Eight classes at each high school, two classes for each of the four grade levels, were selected. Both written and active consent of a parent or legal guardian and student assent was required for participants younger than 18 years. Participants who were 18 years did not require active consent from a parent or guardian. The Institutional Review Board (IRB) of The University of Texas Rio Grande Valley approved the study procedures.

#### 2.3.2. Questionnaire Administration

During the appropriate scheduled 55-min class, participants completed the survey in a school computer laboratory. Participants completed the study measures as part of a larger, anonymous web-based Qualtrics survey. All participants were presented with a statement of assent as a reminder that they could terminate the research at any time or skip any questions that they did not feel comfortable answering without negative implications. Teachers were not in the computer laboratory during this time, and instead two members of the research group were present. Following completion of the survey, participants in each class were entered into a random draw to win two $20.00 gift cards. At the conclusion of the survey, participants were debriefed and given information for The University of Texas Rio Grande Valley Psychology Clinic where they could have confidential access to care.

#### 2.3.3. Statistical Analysis

First, descriptive statistics were computed for demographic variables and prevalence of physical TDV was assessed for three categories: when all four CADRI items were included, when only the minor/moderate severity items were included, and when only the most severe item was included. Second, chi-square statistics were calculated to assess for statistically significant differences in prevalence of TDV perpetration within gender. Third, bivariate correlation analyses were used to evaluate the association between all demographic variables (i.e., age, grade, father and mother’s highest level of education, etc.), study variables and TDV perpetration for each category. Fourth, a series of models were created from binary logistic regression analyses to examine possible predictors associated with perpetrating violence at different severity versus perpetrating no violence. The analyses were conducted separately for males and females so as to determine which variables were significant predictors of physical TDV perpetration at each level of TDV severity for each gender. The demographic variables that were significant (i.e., *p* < 0.05) were included in all adjusted logistic regression analyses. Three dependent variables were used for each set of models. The first dependent variable represented perpetration of any type of physical TDV (i.e., any of the four items), the second dependent variable represented perpetration of only minor/moderate physical TDV perpetration (i.e., three items, excluding kicking, hitting, or punching), and the third dependent variable represented perpetration of only the most severe item (i.e., kicking, hitting, or punching). Missing data were assumed to be missing completely at random, and incomplete cases in each analysis were excluded. All statistical analyses were conducted using SPSS version 25 software [56].

## 3. Results

### 3.1. Descriptive Statistics

From the total sample of adolescents who consented to the research study, 53.3% (*n* = 437) indicated being in a dating relationship in the last twelve months. Of these, 30 adolescents reported being in a same-sex relationship. A response rate of 92% was obtained. Demographic characteristics of the study sample are provided in Table 1.

#### 3.1.1. Prevalence of TDV Perpetration

From the 30 adolescents (female 15, male 15) who reported being in a same-sex relationship, 13.3% (*n* = 4, female 3, male 1) reported perpetrating physical TDV. With regard to adolescents in heterosexual relationships, Table 2 provides the frequency with which both males and females endorsed the four items of physical TDV perpetration and the prevalence of each of the two categories of severity. Among the 407 participants in heterosexual relationships and accounting for missing data, 251 adolescents reported no physical TDV perpetration, 46 reported minor/moderate perpetration of TDV, and 27 reported perpetration of severe TDV. Female adolescents reported perpetrating more violence than their male counterparts when all four items were taken into consideration: slightly less than double the females reported perpetrating violence when compared to males (27.7% vs. 15.7%). For category 1, which included minor to moderate violence, again, slightly nearly three times the number of females reported perpetrating violence at this level when compared to males (20.0% vs. 7.1%; female *n* = 37; male *n* = 9). For category 2, severe violence, the number of males and females reporting kicking, hitting, or punching was very similar (*n* = 13 vs. *n* = 14, respectively). Nonetheless, a higher percentage of males reported severe physical TDV perpetration compared to females (8.7% vs. 7.6%, respectively) which was not significantly different (*p* > 0.05).

Further, there were significant differences in physical TDV perpetration when examining all four items (*χ2* (1) = 6.563, *p* = 0.010). In particular, female adolescents were more likely to push, shove, or shake their partner (*χ2* (1) = 13.713, *p* = 0.003) and throw something at their partner (χ*2* (1) = 10.019, *p* = 0.018), compared with male adolescents. With regard to physical TDV perpetration at category 2 (i.e., minor/moderate severity items), there was a significant difference across gender (*χ2* (1) = 11.965, *p* = 0.001). However, there were no statistically significant differences across gender with respect to slapping or kicking, hitting, or punching perpetration (*p* > 0.05).

We further analyzed those adolescents who endorsed perpetration of any type of physical TDV by comparing the rates of minor/moderate (i.e., category 1) to severe (i.e., category 2) physical TDV perpetration. Of those males who reported any type of violence perpetration, 59.1% reported perpetrating severe violence and 41% reported perpetrating minor/moderate violence. Of those females who reported any type of violence perpetration, 27% reported perpetrating severe violence and 72% reported perpetrating minor/moderate violence. These differences were statistically significant (*χ2* (1) = 6.60, *p* = 0.010).

In addition, Table 3 provides the frequency of overall physical TDV victimization and the prevalence at each of the two categories of severity. Rates of overall victimization were similar for males (27.9%) and females (21.2%), and these differences were not statistically significant (*p* > 0.05). Further, of those males who reported any type of victimization, 67% reported being victims of severe violence and 33% reported being victims of minor/moderate violence. Of the females who reported being victims of physical TDV, 18% reported being victims of severe violence and 82% reported being victims of minor/moderate violence. Again, these differences were statistically significant (*χ2* (1) = 18.96, *p* < 0.001).

#### 3.1.2. Bivariate Correlations

Table 4 and Table 5 display the correlations among study variables at (1) physical TDV at all four items, (2) category 1: minor/moderate items, and (3) category 2: the severe item, for males and females, respectively. All three variables were calculated as binary. For males, age, grade, and father’s highest level of education were positively and significantly associated with physical TDV of all four items and both category 1 and 2. There was a significant positive association between primary language spoken at home and physical TDV at all four physical TDV items and at category 1. These associations were not present for category 2. In addition, dismissive attachment was also significantly and positively associated with physical TDV at category 1 and characteristic of the most severe level of violence, ACEs were significantly and positively associated with category 2.

For females, the demographic variables (i.e., age, grade, primary language spoken at home, mother’s highest level of education) were not positively associated with physical TDV at any of the three variables. Characteristic of all three variables, there was a positive and significant relationship with ACEs and fearful attachment. There was a significant and positive association between ACEs, the four items of physical TDV perpetration, and the most severe item (i.e., category 2). This association was not seen in category 1. In addition, characteristic of only physical TDV perpetration when assessing all four items, DERS was significantly correlated in the positive direction. With regard to impulsivity, there was a significant and positive association with physical TDV perpetration at all four items and the minor/moderate category. Taken together, the differing associations between types of physical TDV perpetration and study variables further demonstrate that taking severity of violence into consideration yields different results.

### 3.2. Prediction of Physical TDV Perpetration Using All Four Items

First, results from the binary logistic regression assessing the relationship between ACEs, exposure to interparental violence and physical TDV perpetration (hypothesis 1) using all four CADRI items indicated that age and exposure to interparental violence were significant predictors of TDV perpetration for males (B = 1.095, *p* = 0.001; B = 2.816, *p* = 0.003, respectively; Table 6). The full model explained between 27.5% (Cox & Snell R^2^) and 48.0% (Nagelkerke R^2^) of the variance. For females, ACEs, and not interparental violence, were a significant predictor of TDV perpetration (B = 4.025, *p* = 0.003).

Second, when the association between attachment and physical TDV perpetration was assessed, neither dismissing attachment in males (hypothesis 2) nor fearful attachment in females (hypothesis 3) was a significant predictor of physical TDV perpetration (*p* > 0.05).

Third, results from the binary logistic regressions assessing the moderation of emotion dysregulation and impulsivity on the association between ACEs, exposure to interparental violence and TDV perpetration (hypothesis 4), indicated that for males there were no significant interactions (*p* > 0.05) showing no moderation effect. However, for females, the interactions between ACEs and emotion dysregulation, and ACEs and impulsivity were statistically significant (B = 0.028, *p* = 0.015; B = 2.803, *p* = 0.002, respectively). This showed the moderation effect of DERS and impulsivity on the association between ACEs, exposure to interparental violence and TDV perpetration. The moderation findings indicate that female adolescents who experienced adverse events in childhood were more likely to perpetrate physical TDV (when assessing all four items) if they also experienced higher emotional dysregulation. Further, female adolescents who experienced adverse events in childhood were more likely to perpetrate physical TDV if they reported being more impulsive. The full model explained between 32.4% (Cox & Snell R^2^) and 50.2% (Nagelkerke R^2^) of the variance.

### 3.3. Prediction of Physical TDV Perpetration of Minor/Moderate Items

First, results from the binary logistic regression assessing the relationship between ACEs, exposure to interparental violence and physical TDV perpetration (hypothesis 1) for category 1 indicated that age and exposure to interparental violence were significant predictors of TDV perpetration for males (B = 2.692, *p* = 0.026; B = 7.649, *p* = 0.048, respectively; Table 7). The full model explained between 21.6% (Cox & Snell R^2^) and 59.8% (Nagelkerke R^2^) of the variance. For females, neither ACEs nor interparental violence were significant predictors of TDV perpetration (*p* > 0.05).

Second, when the association between attachment and physical TDV perpetration was assessed for category 1, dismissing attachment in males (hypothesis 2) was significant (B = 0.241, *p* = 0.037). However, fearful attachment in females (hypothesis 3) was not a significant predictor of physical TDV perpetration (*p* > 0.05).

Third, results from the binary logistic regressions assessing the moderation of emotion regulation and impulsivity on the association between ACEs, exposure to interparental violence and TDV perpetration (hypothesis 4), indicated that for males there were no significant interactions (*p* > 0.05) showing no moderation effect. However, for females, the interactions between ACEs and emotion regulation and ACEs and impulsivity were statistically significant (B = 0.038, *p* = 0.011; B = 3.207, *p* = 0.009, respectively). This showed the moderation effect of DERS and impulsivity on the association between ACEs and TDV perpetration. The moderation findings indicate that female adolescents who experienced adverse events in childhood were more likely to perpetrate physical TDV (when assessing only the moderate items) if they also experienced higher emotional dysregulation. Further, female adolescents that experienced adverse events in childhood were more likely to perpetrate minor/moderate physical TDV if they reported being more impulsive. In addition, the direct effect of emotion dysregulation was also significant (B = 0.055, *p* = 0.046). The full model explained between 18.1% (Cox & Snell R^2^) and 39.1% (Nagelkerke R^2^) of the variance.

### 3.4. Prediction of Physical TDV Perpetration Using Only Severe Item

First, results from the binary logistic regression assessing the relationship between ACEs, exposure to interparental violence and physical TDV perpetration (hypothesis 1) of the most severe item (i.e., category 2) indicated that age, father’s highest level of education, and exposure to interparental violence were significant predictors of TDV perpetration for males (B = 0.942, *p* = 0.010; B = 0.631, *p* = 0.050; B = 3.065, *p* = 0.006, respectively; Table 8). The full model explained between 26.6% (Cox & Snell R^2^) and 52.7% (Nagelkerke R^2^) of the variance. For females, neither ACEs nor interparental violence were significant predictors of TDV perpetration of the most severe item (*p* > 0.05).

Second, when the association between attachment and physical TDV perpetration was assessed, dismissing attachment in males (hypothesis 2) was not significant (*p* > 0.05). However, fearful attachment in females (hypothesis 3) was a significant predictor of physical TDV perpetration of the most severe item (B = 0.119, *p* = 0.047).

Third, results from the binary logistic regressions assessing the moderation of emotion dysregulation and impulsivity on the association between ACEs, exposure to interparental violence and TDV perpetration (hypothesis 4), indicated that for both males and females there were no significant interactions (*p* > 0.05), showing no moderation effect of impulsivity or emotion regulation.

## 4. Discussion

Findings reveal that at least in the Rio Grande Valley, adolescent females report more perpetration of dating violence at minor and moderate levels of violence than males, but not at levels of severe violence where reports are practically equal. Furthermore, when the severity of violence perpetration is evaluated taking into consideration the perpetration at different severity levels within gender, male perpetration of severe violence is substantially higher than that of females. That is, of the total number of males admitting to perpetrating violence, 59 percent admit to perpetrating severe violence, whereas only 27 percent of the females perpetrating violence report perpetrating severe violence. Similarly, there are no differences in reported overall victimization rates between males and females. However, results are different with respect to reported severity of victimization, where female adolescents report more minor to moderate victimization and males report more severe victimization. That is, of the adolescents reporting perpetration and victimization, males are reporting both more severe perpetration and more severe victimization. These findings underscore the need to take the context of the violence into consideration both in studies assessing differential rates of adolescent dating violence perpetration by gender as well as programs designed to address perpetration of dating violence. It is important to note that all violence is not equal. In this study, the majority of the violence perpetrated by females is of minor to moderate severity, whereas the majority of the violence perpetrated by males is moderate to severe. It would be important for studies to report rates of violence by severity levels and not just overall perpetration of violence since this practice can be misleading and does not allow for the development of adequate intervention programs. Again, as in studies assessing intimate partner violence, one size does not appear to fit all [57].

It is also important to differentiate between continuous and categorical assessment of violence perpetration. When perpetration is calculated using a continuous measure, i.e., the sum of perpetration items’ scores, females are found to perpetrate significantly more violence than males. However, when participants are categorized as to whether they perpetrated violence or not, without consideration of a total violence perpetration score these differences are reduced. Again, a continuous approach appears to contribute to misleading results and produces rates of perpetration that are significantly higher in females, but when assessed categorically they are not. Approaches that utilize the sum of scores on items of perpetration facilitate some statistical analyses like validity studies, but in reality, mask important information that is valuable for clinical interpretation of the results. In these approaches, a few individuals contribute disproportionately to the total score. However, categorical analysis informs as to the number of individuals who perpetrate violence and because an individual is only counted once, that individual does not contribute disproportionately. Finally, for clinical or intervention purposes, one generally wants to know if an individual should be selected for prevention of treatment, thus that pushes one to a categorical decision.

The issue of categorical versus dimensional approaches to assessment have been debated for decades, and the issue has received most recent diagnostic attention regarding personality disorder assessment, with a trial dimensional approach now under evaluation [58]. In the case of personality disorders, a multidimensional trait model, rather than focusing on one categorical label, the alternative model focuses on all five personality dimensions. The general argument is that most psychopathologies are best conceptualized as continuous constructs. However, in the case of personality disorders, the dimensional approach has not yet proven to be useful for formal diagnosis and insurance reimbursement. One of the main arguments in favor of a dimensional approach is the ability to obtain larger validity coefficients with a greater range and variability of scores. For example, Shankman et al. (2018) examined whether severity dimensions have better psychometric properties (internal consistency, test–retest reliability, and concurrent and predictive validity) than categorical diagnoses [59]. Participants (*n*= 234) were recruited from the community and from clinics. Dimensional severity scales were created from an adapted version of the SCID for eleven different disorders, and the symptom severity scales demonstrated significant incremental validity over and above categorical diagnoses for both current and prospective outcomes. Moreover, a meta-analysis has shown that dimensionally assessed psychopathology has 15% greater reliability and 37% greater validity [60]. However, in this sample, when regression analyses were conducted using continuous measures of violence versus the categorical measures of violence, the analyses were not different. The most reasonable conclusion regarding selection of use of dichotomous versus continuous measurement of dating aggression is to analyze data using both approaches, and in turn select one or the other based on theoretical and pragmatic reasons.

Findings from the present study also revealed that experience of adverse events in childhood (ACEs), witnessing interparental violence, emotional regulation difficulties, impulsivity, and attachment are all significantly related to perpetration of dating violence by adolescents. However, these relationships are different in males and females, for different levels of violence. Differences were found when the analyses to identify variables that are related to violence perpetration focused on global assessment of violence (using all four items) as opposed to differentiating between the contribution of these variables to perpetration of minor to moderate violence versus severe violence.

For males but not for females, age was positively correlated with perpetration of violence against a partner in this sample of Hispanic adolescents from the Rio Grande Valley in Texas, and in this sample the highest rates were at 18 years. In a longitudinal sample of 973 adolescents aged 13–19, partner aggression was found to be highest at about 17–18 years [61]. The trajectory increased to a peak and then declined. The peak age for moderate IPV was 17.1 years and the peak age for severe IPV was 16.3 years. Relatedly, in a cross-sectional sample of 2416 Spanish adolescents aged 16–20 years, Munoz-Rivas, Grana, O’Leary & Gonzalez (2007) found that physical aggression decreased across the age groups, with the numerically highest age being 17 but health consequences became more severe with age [62]. Large-sample longitudinal studies in different geographical areas of the country using similar measures and perhaps at least two measures of dating aggression are needed before one can know when partner aggression peaks.

In the global analyses in males, age and witnessing interparental violence are found to predict perpetration of violence. That is, older males with exposure to interparental violence are more likely to perpetrate violence in their dating relationship. However, these results do not hold nor apply equally to different levels of violence. When we consider perpetration of minor and moderate violence in males, age, witnessing interparental violence, and dismissing attachment are predictive of perpetration. That is, older males with histories of witnessing interparental violence and who score higher on dismissive attachment are more likely to perpetrate minor to moderate violence in their emerging dating relationship. Finally, when we consider only perpetration of severe aggression, age, father’s highest level of education and witnessing interparental violence are predictive of perpetration. That is, older adolescent males who have witnessed interparental violence and whose fathers have a higher level of education are more likely to perpetrate severe violence in their dating relationships. When violence is assessed globally in females, experiencing adverse events in childhood predicts perpetration of dating violence and this relationship is moderated by both difficulties with emotional regulation and impulsivity. Namely, adolescent females who experience adverse events in childhood and have more difficulties with emotional regulation and impulsivity are more likely to perpetrate violence in their dating relationships. This relationship was also found in females who perpetrate minor to moderate violence. However, only fearful attachment is predictive of severe violence in females. Specifically, adolescent females who have higher level of fearful attachment difficulties are more likely to perpetrate severe violence.

ACEs are associated with perpetration of minor to moderate violence in females. That is, female adolescents who experience a higher number of adverse events in childhood are more likely to perpetrate minor/moderate violence in their dating relationships irrespective of the severity level. This relationship appears to be moderated by emotional dysregulation and impulsivity at low levels of violence perpetration. Fearful attachment is particularly important at the most severe level. Females who develop insecure attachment are more likely to perpetrate more severe violence. It could be that relationship attachment-related events such as instances of perceived abandonment and/or jealousy issues are particularly anxiety provoking for females with insecure or fearful attachment issues, and lead to perpetration of severe violence. In males, age and witnessing interparental violence are related to perpetration of violence at both minor/moderate and severe levels. That is, older adolescents who have witnessed interparental violence are more likely to perpetrate violence in their dating relationships. Additionally, dismissive attachment is associated with minor to moderate violence and father’s highest level of education with severe violence perpetration. Surprisingly, emotion regulation skills do not appear to be related to male perpetration of dating violence. It is, however, possible that this finding could be related to the male reporting style and that males could be responding in socially desirable ways and minimizing any difficulties with emotional regulation and impulsivity [63]. Father’s educational level and income are correlated, and income is in turn related to perpetration of violence at severe levels. One could speculate that this could be related to a lack of tolerance to adversity. It would be important for subsequent research to explore this possibility. While experiencing adverse events in childhood and witnessing interparental violence appear to increase the probability that an adolescent will perpetrate physical violence in their dating relationships, it does not do so for all adolescents. It is clear from the findings presented that a reasonable percentage of adolescents experience ACEs and witness interparental violence and they themselves do not perpetrate violence in their relationships. Given the findings reported, it is reasonable to assume that those female adolescents who also have difficulties with emotional regulation, impulsivity, and attachment are more likely to go on to perpetrate violence. It is possible that some adolescents who experience adverse childhood events and witness interparental violence are exposed to corrective emotional experiences which prevent them from eventually perpetrating violence in their intimate relationships. Some adolescents appear more resilient to trauma than others [64]. It is important for future research to identify factors that would promote this resiliency in order to include them in prevention strategies.

The results presented herein suggest possible avenues for primary prevention strategies that could target adolescents prior to the age when they are likely to get involved in romantic relationships, towards the end of their latency and the beginning of the genital developmental stage. Dating violence prevention programs should promote the development of optimal emotional regulation and impulse control skills with an emphasis on affective relationships, especially in females. With respect to adolescent males, the findings from this study would suggest that processing their experience of witnessing interparental violence is particularly relevant, as well as discussions of constructive communication and problem-solving skills in romantic relationships. In addition, focusing on attachment in intimate relationships, by exploring both the female and male adolescents’ attachment style, events that had a strong impact on its development, and discussion of development of secure attachment styles in their subsequent relationships, would likely help reduce the emergence of violence in their emerging dating relationships.

Finally, the population on which the present study’s findings were based is drawn from the Rio Grande Valley, which is 92% Hispanic. Overall rates of violence perpetration are within the range of findings from other studies from other parts of the USA as well as other countries, but on the low side [15]. Unlike other studies, the data were analyzed with respect to the severity of violence by gender, showing that overall, males report that when they perpetrate violence, they tend to perpetrate more severe violence than when females perpetrate violence. They also report that when they experience victimization, they experience more severe victimization than females. We do not know whether this finding is specific to the Hispanic population of the Rio Grande Valley or if it is representative of other populations as well since most studies only report overall levels of violence perpetration. It would be important for other studies that have reported overall levels of higher violence perpetration by females to reanalyze their data with respect to different rates of perpetration at different severity levels and for subsequent studies to report analyses based on severity of TDV. This would allow for an understanding of the context of the violence and development of more specific interventions. In summary, results from the present study found that experiencing adverse events in childhood increases the probability that a female adolescent will perpetrate violence in their own dating relationship, and that this is moderated by emotion regulation ability, impulsivity, and attachment, and that witnessing interparental violence has particularly adverse effects in males.

### Study Limitations

The findings of the present study should be interpreted considering several limitations. First, retrospective recall of childhood maltreatment and ACEs can lead to bias in reporting. Second, the present study identifies that the selection of the high schools participating in research is convenience-based, thus it creates a selection risk to the study’s internal validity. Third, given that all data were collected at the same time, it is important to be cautious about inferring causality. The present study assumes that the contextual variables analyzed precede the perpetration of violence. Fourth, the creation of severity scores was based on the nature of the act (e.g., shoving vs. hitting) which is one aspect of severity of abuse as noted by prior literature [65]. Fourth, given that the purpose of the current study was to identify gender differences at different levels of physical abuse, the samples were divided by gender when conducting logistic regressions. An advantage of this analytic strategy is that it allows the identification of gender differences at each level of physical abuse in a straightforward manner; however, subsetting the sample and conducting multiple analyses separately can affect the power of the statistical tests. In the current study, we tried to clarify these statistical confounds by reporting the odds ratio of each predictor as an effect-size measure, given that it is less sensitive to the sample sizes in comparison to the *p*-values [66]. Given the results presented in Tables 6 through 9, most odds ratios of the significant study variables indicated medium to large effects (odd ratios > 3.47, according to the criteria proposed by Chen et al., 2010) [66]. Thus, we think these results can be considered as additional evidence that supports the conclusions, regardless of the power of the analyses. However, there are still a few predictors in our analyses that only have small effect sizes (e.g., effects of DERS and fearful attachment in Table 7 and Table 8, respectively, odd ratios < 1.68). Future studies are needed to further confirm the effects of these predictors on physical TDV. Fifth, the data was obtained via self-report and are subject to self-report bias. It could be that males were underreporting, with respect to perpetration of violence, and more defensive in their responses to the emotional dysregulation and attachment questionnaires. While both males and females underreport partner violence, males evidence more underreporting of partner violence than females [67]. However, in this study, with respect to perpetration of violence, it is unlikely that the males were underreporting given that their reports of perpetration matched those of female reports of victimization. However, it is possible that the failure to find association between difficulties with emotional regulation and perpetration of violence could be due to a tendency to minimize difficulties and respond in a socially desirable manner. It would be a good idea to include a measure of social desirability such as the Marlow–Crowne [68] in future studies to control for this possibility.

## 5. Conclusions

Findings reveal that at least in the Rio Grande Valley, Texas, adolescent females report more perpetration of dating violence at minor and moderate levels of violence than males, but not at levels of severe violence where reports are practically equal. Furthermore, when the severity of violence perpetration is evaluated, taking into consideration the perpetration at different severity levels within gender, male perpetration of severe violence is substantially higher than that of females. Although there are no differences in reported overall victimization rates between males and females, results are different with respect to the reported severity of victimization, where female adolescents report more minor to moderate victimization and males report more severe victimization. Findings from the present study also revealed that experience of adverse events in childhood (ACEs), witnessing interparental violence, emotional regulation difficulties, impulsivity, and attachment are all significantly related to perpetration of dating violence by adolescents. However, these relationships are different in males and females, for different levels of violence.

The results presented herein suggest that dating violence prevention programs should target development of optimal emotional regulation and impulse control skills with an emphasis on affective relationships, especially in females. With respect to adolescent males, the findings from this study would suggest that processing their experience of witnessing interparental violence is particularly relevant, alongside discussion of constructive communication and problem-solving skills in romantic relationships. In addition, focusing on attachment in intimate relationships would likely help reduce the emergence of violence in their developing dating relationships. The importance of assessing perpetration of violence both categorically and dimensionally was highlighted.

## Figures and Tables

**Table 1:** Demographic statistics of the study sample (*N* = 407).

Variable	N	Percentage of Sample
Gender		
Male	189	46.4%
Female	218	53.6%
Age		
14	67	16.5%
15	75	18.4%
16	90	22.1%
17	97	23.8%
18	78	19.2%
Grade		
9	72	17.7%
10	106	26.0%
11	129	31.7%
12	100	24.6%
Race/Ethnicity		
Hispanic/Latino	360	88.5%
Other	47	11.5%
Primary language spoken at home		
Spanish	196	48.2%
English	208	51.1%
Other	3	0.7%

**Table 2:** Prevalence of physical TDV perpetration by item and three categories.

	Males (*n* = 149)	Females (*n* = 184)
	% (*n*)	% (*n*)
1. I pushed, shoved, or shook	8.7 (13)	15.8 (29)
2. I threw something	6.0 (9)	14.1 (26)
3. I slapped or pulled hair	8.7 (13)	13.6 (25)
4. I kicked, hit, or punched	8.7 (13)	7.6 (14)
**Categories**		
^a^ All four items	15.7 (22)	27.7 (51)
^b^ Category 1: Minor/Moderate (items 1–3)	7.1 (9)	21.8 (37)
^c^ Category 2: Severe (item 4)	8.7 (13)	7.6 (14)

^a^*n* = 140 for males; *n* = 184 for females.; ^b^ *n* = 127 for males; *n* = 170 for females; ^c^ *n* = 149 for males; *n* = 184 for females.

**Table 3:** Prevalence of physical TDV victimization by item and three categories.

	Males (*n* = 149)	Females (*n* = 184)
	% (*n*)	% (*n*)
1. He/she pushed, shoved, or shook me	12.1 (18)	12.0 (22)
2. He/she threw something at me	11.4 (17)	8.7 (16)
3. He/she slapped or pulled my hair	14.1 (21)	8.7 (16)
4. He/she kicked, hit, or punched me	17.4 (26)	3.8 (7)
**Categories**		
^a^ All four items	27.9 (39)	21.2 (39)
^b^ Category 1: Minor/Moderate (items 1–3)	11.4 (13)	18.1 (32)
^c^ Category 2: Severe (item 4)	17.4 (26)	3.8 (7)

^a^*n* = 140 for males; *n* = 184 for females.; ^b^ *n* = 114 for males; *n* = 177 for females; ^c^ *n* = 149 for males; *n* = 184 for females.

**Table 4:** Bivariate correlations among study variables for males.

	1	2	3	4	5	6	7	8	9	10
Males (*n* = 189)										
1. Physical TDV (All 4 items)	-	0.30 **	0.30 **	0.19 *	0.20 *	0.15	0.26 **	0.17	0.10	0.01
2. Age		-	0.64 **	0.04	−0.07	0.07	−0.07	0.07	0.06	−0.08
3. Grade			-	0.07	−0.11	0.15	−0.03	0.13	−0.04	−0.03
4. Language spoken at home				-	−0.13	−0.19 *	−0.04	−0.01	0.02	−0.16 *
5. Father’s level of education					-	0.12	0.01	0.27 **	−0.10	−0.02
6. ACEs						-	0.41 **	0.23 **	0.21 *	0.35 **
7. Interparental violence							-	0.01	0.25 **	0.09
8. Dismissing attachment								-	0.16	0.15
9. Impulsivity									-	0.28 **
10. DERS										-
1. Physical TDV (Minor/Moderate)	-	0.23 **	0.22 *	0.23 **	0.05 *	0.02	0.06 *	0.25 **	0.11	0.07
2. Age		-	0.60 **	0.06	−0.09	−0.01	−0.17	0.09	0.01	−0.06
3. Grade			-	0.11	−0.14	0.09	−0.05	0.16	−0.09	0.02
4. Language spoken at home				-	−0.13	−0.18 *	−0.06	−0.09	0.03	−0.19 *
5. Father’s level of education					-	0.05	−0.04	0.30 **	−0.12	−0.03
6. ACEs						-	0.47 **	0.32 **	0.17	0.49 **
7. Interparental violence							-	0.10	0.24 **	0.20 *
8. Dismissing attachment								-	0.17	0.14
9. Impulsivity									-	0.35 **
10. DERS										-
1. Physical TDV (Severe)	-	0.18 *	0.17 *	0.08	0.26 **	0.21 *	0.33 **	0.01	0.04	0.08
2. Age		-	0.64 **	0.04	−0.07	0.07	−0.07	0.07	0.06	−0.08
3. Grade			-	0.07	−0.11	0.15	−0.03	0.13	−0.04	−0.03
4. Language spoken at home				-	−0.13	−0.19 *	−0.04	−0.01	0.02	−0.16 *
5. Father’s level of education					-	0.12	0.01	0.27 **	−0.10	−0.02
6. ACEs						-	0.41 **	0.23 **	0.21 *	0.35 **
7. Interparental violence							-	0.01	0.25 **	0.09
8. Dismissing attachment								-	0.16	0.15
9. Impulsivity									-	0.28 **
10. 1DERS										-

* *p* < 0.05; ** *p* < 0.01; *** *p* < 0.001.

**Table 5:** Bivariate correlations among study variables for females.

	1	2	3	4	5	6	7	8	9	10	11	12
Females (*n* = 218)												
1. Physical TDV (All 4 items)	-	0.10	0.07	−0.02	−0.10	0.23 **	0.19 *	0.32 **	0.36 **	0.27 **	0.27 **	0.23 **
2. Age		-	0.80 **	−0.00	−0.10	0.11	0.02	0.22 **	0.21 *	0.04	0.03	0.18 *
3. Grade			-	−0.03	−0.11	0.05	−0.03	0.17 *	0.07	−0.04	−0.05	0.07
4. Language spoken at home				-	−0.29 **	−0.02	−0.09	−0.16 *	−0.12	−0.09	−0.08	−0.13
5. Mother’s level of education					-	−0.19 *	0.02	−0.22 **	−0.10	0.06	−0.11	−0.07
6. ACEs						-	0.40 **	0.37 **	0.45 **	0.10	0.22 **	0.36 **
7. DERS							-	0.37 **	0.66 **	0.37 **	0.18 *	0.55 **
8. Fearful attachment								-	0.16	−0.05	−0.02	0.14
9. Impulsivity									-	0.35 **	0.22 *	0.84 **
10. Perseverance										-	0.66 **	0.03
11. Lack of premeditation											-	−0.08
12. Positive urgency												-
1. Physical TDV (Minor/Moderate)	-	0.04	0.01	−0.01	−0.10	0.12	0.14	0.24 **	0.28 **	0.26 **	0.24 **	0.23 **
2. Age		-	0.77 **	−0.00	−0.06	0.07	0.02	0.18 *	0.16	0.06	0.02	−0.04
3. Grade			-	−0.05	−0.07	0.01	−0.03	0.14	0.032	−0.03	−0.06	−0.12
4. Language spoken at home				-	−0.21 **	−0.04	−0.10	−0.13	−0.09	−0.11	−0.07	−0.08
5. Mother‘s level of education					-	−0.13	0.02	−0.22 **	−0.05	0.06	−0.10	−0.06
6. ACEs						-	0.40 **	0.35 **	0.40 **	0.10	0.18 *	0.18 *
7. DERS							-	0.38 **	0.60 **	0.33 **	0.12	0.40 **
8. Fearful attachment								-	0.14	−0.04	−0.05	−0.02
9. Impulsivity									-	0.32 **	0.16	0.75 **
10. Perseverance										-	0.65 **	0.02
11. Lack of premeditation											-	−0.20 *
12. Positive urgency												-
1. Physical TDV (Severe)	-	0.14	0.12	0.00	−0.02	0.26 **	0.10	0.22 **	0.17	0.08	0.15	0.18 *
2. Age		-	0.80 **	−0.00	−0.10	0.11	0.02	0.22 **	0.21 *	0.04	0.03	0.18 *
3. Grade			-	−0.03	−0.11	0.05	−0.03	0.17 *	0.07	−0.04	−0.05	0.07
4. Language spoken at home				-	−0.29 **	−0.02	−0.09	−0.16 *	−0.12	−0.09	−0.08	−0.13
5. Mother’s level of education					-	−0.19 *	0.02	−0.22 **	−0.10	0.06	−0.11	−0.07
6. ACEs						-	0.40 **	0.37 **	0.45 **	0.10	0.22 *	0.36 **
7. DERS							-	0.37 **	0.66 **	0.37 **	0.18 *	0.55 **
8. Fearful attachment								-	0.16	−0.05	−0.02	0.14
9. Impulsivity									-	0.35 **	0.22 *	0.84 **
10. Perseverance										-	0.66 **	0.03
11. Lack of premeditation											-	−0.08
12. Positive urgency												-

* *p* < 0.05; ** *p* < 0.01; *** *p* < 0.001.

**Table 6:** Logistic regression: Relationship between study variables and physical TDV perpetration using all four items of the CADRI physical abuse subscale in males and females *.

Variables Related to Hypothesis	B (OR)	95% CI for OR
**MALES**		
*Control Variables*		
**Age**	1.095 (2.989) **	[1.537, 5.814]
**Father’s level of education**	0.413 (1.512)	[0.877, 2.606]
*Study Variables*		
**Adverse Childhood Experiences**	0.005 (1.005)	[0.735, 1.373]
**Interparental violence**	2.816 (16.718) **	[2.605, 107.302]
**Dismissing attachment**	0.053 (1.054)	[0.974, 1.142]
−2 Log likelihood	55.919	
**FEMALES**		
*Study Variables*		
**Adverse Childhood Experiences**	4.025 (0.018) **	[0.001, 0.266]
**Interparental violence**	4.412 (0.012)	[0.000, 4.8 × 10^10^]
**DERS**	0.022 (1.022)	[0.981, 1.065]
**Impulsivity**	1.666 (0.189)	[0.010, 3.433]
**Fearful attachment**	0.096 (1.101)	[0.985, 1.230]
**ACEs × DERS**	0.028 (0.972) *	[0.951, 0.994]
**ACEs × Impulsivity**	2.803 (16.486) **	[2.901, 93.698]
−2 Log likelihood	87.525	

* A linear regression analysis was conducted, with a continuous dependent variable including all four items of the CADRI, and the same results were obtained. Note: OR = odds ratio; CI = confidence interval. * *p* < 0.05; ** *p* < 0.01; *** *p* < 0.001.

**Table 7:** Logistic regression: Relationship between study variables and minor/moderate physical TDV perpetration in males and females.

Variables Related to Hypothesis	B (OR)	95% CI for OR
**MALES**		
*Control Variables*		
**Age**	2.692 (14.755) *	[1.380, 157.819]
**Father’s level of education**	1.024 (2.784)	[0.553, 14.022]
*Study Variables*		
**Adverse Childhood Experiences**	1.588 (.204)	[.034, 1.226]
**Interparental violence**	7.649 (2097.92) *	[1.068, 4.12 × 10^6^]
**Dismissing attachment**	0.241 (1.273) *	[1.015, 1.597]
−2 Log likelihood	20.831	
**FEMALES**		
*Study Variables*		
**Adverse Childhood Experiences**	2.904 (0.055)	[0.002, 1.884]
**Interparental violence**	33.609 (0.000)	[0.000, 1.37 × 10^27^]
**DERS**	0.055 (1.056) *	[1.001, 1.115]
**Impulsivity**	2.765 (0.063)	[0.001, 3.365]
**Fearful attachment**	0.085 (1.089)	[0.921, 1.287]
**ACEs × DERS**	0.038 (0.963) *	[0.935, 0.991]
**ACEs × Impulsivity**	3.207 (24.701) **	[2.256, 270.493]
−2 Log likelihood	54.038	

Note: OR = odds ratio; CI = confidence interval. * *p* < 0.05; ** *p* < 0.01; *** *p* < 0.001.

**Table 8:** Logistic regression: Relationship between study variables and severe physical TDV perpetration in males and females.

Variables Related to Hypothesis	B (OR)	95% CI for OR
**MALES**		
*Control Variables*		
**Age**	0.942 (2.564) *	[1.251, 5.258]
**Father’s level of education**	0.631 (1.880)	[0.999, 3.537]
*Study Variables*		
**Adverse Childhood Experiences**	0.200 (1.222)	[0.851, 1.754]
**Interparental violence**	3.065 (21.424) **	[2.420, 189.669]
**Dismissing attachment**	−0.017 (0.983)	[8.93, 1.083]
−2 Log likelihood	42.020	
**FEMALES**		
*Study Variables*		
**Adverse Childhood Experiences**	0.052 (1.053)	[0.700, 1.585]
**Interparental violence**	0.099 (1.104)	[0.102, 11.940]
**DERS**	0.003 (1.003)	[9.63, 1.044]
**Impulsivity**	0.972 (2.643)	[0.114, 61.174]
**Fearful attachment**	0.119 (1.126) *	[1.002, 1.265]
−2 Log likelihood	45.217	

Note: OR = odds ratio; CI = confidence interval. * *p* < 0.05; ** *p* < 0.01; *** *p* < 0.001.
